# Optimal timing of salvage radiotherapy for biochemical recurrence after radical prostatectomy: is ultra-early salvage radiotherapy beneficial?

**DOI:** 10.1186/s13014-016-0671-1

**Published:** 2016-07-30

**Authors:** Satoru Taguchi, Kenshiro Shiraishi, Hiroshi Fukuhara, Keiichi Nakagawa, Teppei Morikawa, Akihiro Naito, Shigenori Kakutani, Yuta Takeshima, Hideyo Miyazaki, Tohru Nakagawa, Tetsuya Fujimura, Haruki Kume, Yukio Homma

**Affiliations:** 1 Department of Urology, Graduate School of Medicine, The University of Tokyo, 7-3-1 Hongo, Bunkyo-ku, Tokyo 113-8655 Japan; 2 Department of Radiology, The University of Tokyo Hospital, 7-3-1 Hongo, Bunkyo-ku, Tokyo 113-8655 Japan; 3 Department of Pathology, Graduate School of Medicine, The University of Tokyo, 7-3-1 Hongo, Bunkyo-ku, Tokyo 113-8655 Japan

**Keywords:** Biochemical recurrence, Salvage, Prostate cancer, Prostatectomy, Radiation, Radiotherapy

## Abstract

**Background:**

The optimal timing of salvage radiotherapy for biochemical recurrence after radical prostatectomy is controversial. In particular, the prognostic significance of salvage radiotherapy delivered before a current definition of biochemical recurrence, i.e. ultra-early salvage radiotherapy, is unclear.

**Methods:**

We reviewed 76 patients with pT2-3N0M0 prostate cancer who underwent salvage radiotherapy for post-prostatectomy biochemical recurrence at the following three timings: ultra-early salvage radiotherapy (*n* = 20) delivered before meeting a current definition of biochemical recurrence (two consecutive prostate-specific antigen [PSA] values ≥0.2 ng/mL); early salvage radiotherapy (*n* = 40) delivered after meeting the definition but before PSA reached 0.5 ng/mL; and delayed salvage radiotherapy (*n* = 16) delivered after PSA reached 0.5 ng/mL. The primary endpoint was failure of salvage radiotherapy, defined as a PSA value ≥0.2 ng/mL. The log-rank test and Cox proportional hazards model were used for univariate and multivariate analyses, respectively.

**Results:**

During the follow-up period (median: 70 months), four of 20 (20 %), nine of 40 (23 %) and seven of 16 (44 %) patients failed biochemically in the ultra-early, early and delayed salvage radiotherapy groups, respectively. On univariate analyses, the outcome of delayed salvage radiotherapy was worse than the others, while there was no significant difference between ultra-early and early groups. Multivariate analysis demonstrated the presence of Gleason pattern 5, perineural invasion and delayed salvage radiotherapy as independent predictors of poorer survival.

**Conclusions:**

No survival benefit of ultra-early salvage radiotherapy was demonstrated, whereas delayed salvage radiotherapy was associated with worse outcome as reported in previous studies. Our results may support the current recommendations that salvage radiotherapy should be undertaken after two consecutive PSA values ≥0.2 ng/mL and before reaching 0.5 ng/mL.

## Background

Approximately 25–35 % of patients develop biochemical recurrence (BCR) after radical prostatectomy (RP) for clinically localized prostate cancer (PC) [1, 2]. Although salvage radiotherapy (SRT) is a standard treatment option for post-RP BCR [3–5], there is currently no consensus regarding its optimal timing. There are several commonly used definitions of BCR; most involve single or multiple prostate-specific antigen (PSA) values of at least ≥0.2 [6], including the current official definition in Japan of two consecutive PSA values ≥0.2 ng/mL [7]. To the best of our knowledge, the value of SRT delivered before meeting these definitions has not been investigated to date. We previously reported that salvage androgen deprivation therapy (ADT) administered before meeting the Japanese definition, referred to as “ultra-early salvage ADT”, achieved a better oncological outcome than ADT administered after patients met the definition in pT2-4 N0 PC [8].

On the other hand, several recent studies have suggested that SRT should be started before PSA levels reach 0.5 ng/mL [9–13]. Notably, Briganti et al. demonstrated that early SRT (eSRT; given at pre-radiation PSA ≤0.5 ng/mL) achieved an oncological outcome equivalent to adjuvant radiotherapy in patients with pT3N0 PC [10].

In this context, the present study aimed to investigate the benefit of SRT given before patients meet a currently used definition [7] (i.e. ultra-early SRT; ueSRT), by comparing the outcomes of patients treated with SRT at different timings after RP.

## Methods

### Patients

This retrospective analysis was approved by the internal institutional review board of Graduate School of Medicine and Faculty of Medicine, The University of Tokyo (approval number: 3124). We reviewed 96 patients who underwent SRT after RP at our institution between 2006 and 2014. Of them, 19 patients who received ADT (neoadjuvant and/or adjuvant: 13; salvage: 6) prior to SRT and a patient who underwent SRT for pN1 disease were excluded. Finally, we retrospectively reviewed 76 patients with pT2-3N0M0 PC who underwent SRT after RP at our institution during this period. Of the 76 patients, 20 (26 %) received ueSRT for increasing PSA but before they met a current definition of BCR (two consecutive PSA values ≥0.2 ng/mL [7]); 40 (53 %) received eSRT after meeting the definition but before PSA reached 0.5 ng/mL; and 16 (21 %) received delayed SRT (dSRT) after PSA reached 0.5 ng/mL. All 20 patients in the ueSRT group had at least one detectable PSA value, although they did not meet the above definition of BCR: In more details, 18 of 20 (90 %) patients started SRT before PSA values reached 0.2 ng/mL; and the remaining 2 (10 %) did it over only a single PSA value of 0.2 ng/mL. PSA doubling time was calculated in patients who had at least three PSA measurements after BCR but before initiating SRT, using all PSA measurements. The calculation assumed first-order kinetics, dividing the natural logarithm of 2 by the slope of the log PSA vs time of PSA measurements for each patient (in months) [14].

### Treatments

The most common surgical procedure of open RP with bilateral obturator lymph node dissection was performed in 59 (78 %) patients. Laparoscopic RP and robot-assisted laparoscopic RP were performed in six (8 %) and 11 (14 %) patients, respectively.

The standard technique for SRT was 3-dimensional conformal radiation therapy (3DCRT) in 61 (80 %) patients, while 15 (20 %) patients were treated with intensity-modulated radiotherapy (IMRT) to cover the prostate bed with reference to preoperative computed tomography and/or magnetic resonance imaging (Table 1). The most common delivered dose was 66 Gy, though a few (6 of 76 [8 %]) patients received 70 Gy by IMRT with a simultaneous integrated boost to suspicious recurrent lesions detected by multi-parametric magnetic resonance imaging in addition to 66 Gy to the prostate bed, as per our ongoing prospective study (UMIN000009823: Dynamic contrast-enhanced magnetic resonance imaging study for biochemical failures after radical prostatectomy). All SRT regimens were given in 1.8–2.0-Gy daily fractions. For pre-radiation studies, computed tomography was used in most patients, whereas six (8 %) who participated in the prospective study mentioned above underwent 3-tesla dynamic contrast enhanced magnetic resonance imaging.

**Table 1:** Patient characteristics

Parameter	Total (*n* = 76)	ueSRT (*n* = 20)	eSRT (*n* = 40)	dSRT (*n* = 16)	*P*
Age at surgery, years, median (IQR)	65 (61–69)	68 (61–72)	65 (61–69)	64 (57–67)	0.0574^a^
Initial PSA, ng/mL, median (IQR)	8.7 (6.4–12.6)	9.2 (6.9–12.6)	8.4 (5.9–13.0)	9.3 (7.2–12.9)	0.6684^a^
Pathological T stage, no. (%):					
T2	37 (49)	9 (45)	21 (53)	7 (44)	0.0834^b^
T3a	35 (46)	10 (50)	19 (48)	6 (38)	
T3b	4 (5)	1 (5)	0 (0)	3 (19)	
Pathological GS, no. (%):					
≤6	12 (16)	3 (15)	6 (15)	3 (19)	0.1533^b^
7	49 (64)	16 (80)	26 (65)	7 (44)	
≥8	15 (20)	1 (5)	8 (20)	6 (38)	
Gleason pattern 5 (including tertiary 5)	27 (36)	5 (25)	14 (35)	8 (50)	0.2959^b^
Extraprostatic extension, no. (%)	37 (49)	10 (50)	18 (45)	9 (56)	0.7416^b^
Lymphovascular invasion, no. (%)	21 (28)	3 (15)	10 (25)	8 (50)	0.0568^b^
Positive surgical margin, no. (%)	57 (75)	17 (85)	28 (70)	12 (75)	0.4493^b^
Seminal vesicle invasion, no. (%)	4 (5)	1 (5)	0 (0)	3 (19)	0.0178*^b^
Perineural invasion, no. (%)	58 (76)	17 (85)	28 (70)	13 (81)	0.3805^b^
SRT technique, no. (%):					
3DCRT	61 (80)	19 (95)	27 (68)	15 (94)	0.0130*^b^
IMRT	15 (20)	1 (5)	13 (33)	1 (6)	
Total dose, no. (%):					
60–65 Gy	10 (13)	3 (15)	5 (13)	2 (13)	0.9646^b^
66 Gy	60 (79)	16 (80)	31 (78)	13 (81)	
70 Gy	6 (8)	1 (5)	4 (10)	1 (6)	
Concomitant ADT, no. (%)	12 (16)	5 (25)	2 (5)	5 (31)	0.0218*^b^
PSA doubling time, months, median (IQR)	8.4 (4.6–20.1)	–†	12.7 (6.2–25.1)	5.9 (3.5–14.3)	–
Median follow-up, months (IQR)	70 (48–95)	74 (57–99)	73 (43–83)	58 (41–99)	0.5386^a^

*SRT* salvage radiotherapy, *ueSRT* ultra-early salvage radiotherapy, *eSRT* early salvage radiotherapy, *dSRT* delayed salvage radiotherapy, *IQR* interquartile range, *PSA* prostate-specific antigen, *GS* Gleason score, *3DCRT* 3-dimensional conformal radiation therapy, *IMRT* intensity-modulated radiotherapy, *ADT* androgen deprivation therapy

*Statistically significant; ^a^one-way ANOVA; ^b^Pearson’s χ^2^ test

†In the ueSRT group, PSA doubling time was only calculable in two patients (3.9 and 5.6 months)

### Endpoints and statistical analysis

The primary endpoint was failure of SRT, defined as a PSA value ≥0.2 ng/mL after PSA nadir following SRT [9]. Secondary endpoints were clinical metastasis and cancer-specific mortality. Associations of various clinicopathological factors, including timing of SRT, with failure-free survival were assessed. Survival time was defined as the time (months) between RP and failure of SRT or last follow-up [10]. The log-rank test and Cox proportional hazards model were used for univariate and multivariate analyses, respectively. All statistical analyses were performed using JMP Pro version 11.0.0 (SAS Institute, Cary, NC, USA). A value of *P* < 0.05 was considered significant. Follow-up information was obtained as of August 2015.

## Results

**Fig. 1:**
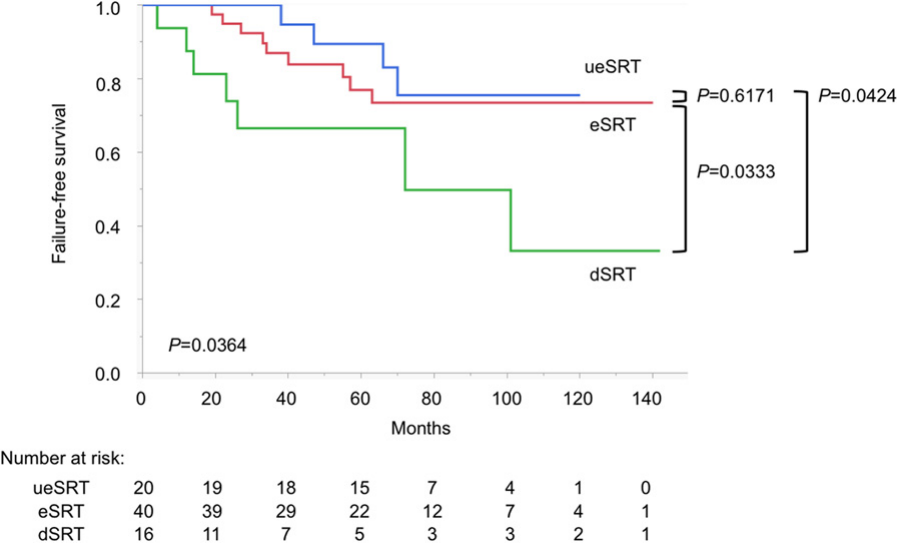
Kaplan–Meier curves depicting failure-free survival according to three timings of SRT (log-rank test: *P* = 0.0364)

**Fig. 2:**
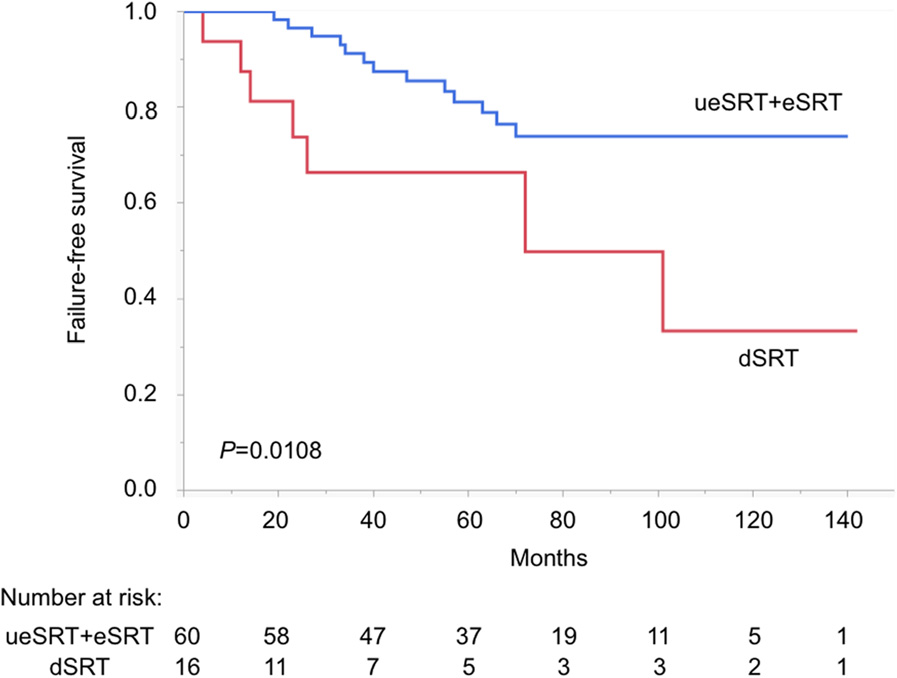
Kaplan–Meier curves depicting failure-free survival of ueSRT + eSRT vs dSRT (log-rank test: *P* = 0.0108)

**Fig. 3:**
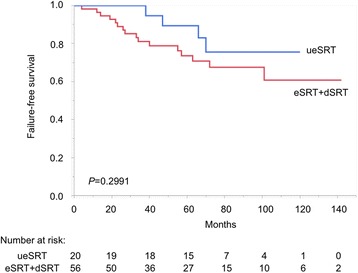
Kaplan–Meier curves depicting failure-free survival of ueSRT vs eSRT + dSRT (log-rank test: *P* = 0.2991)

The patient characteristics of the whole population (*n* = 76) and according to the timing of SRT are summarized in Table 1. During the follow-up period (median: 70 months; interquartile range: 48–95 months), four of 20 (20 %), nine of 40 (23 %) and seven of 16 (44 %) patients developed failure of SRT in the ueSRT, eSRT and dSRT groups, respectively. The outcome of dSRT was significantly worse than the others (log-rank test: *P* = 0.0424 for ueSRT; *P* = 0.0333 for eSRT), while there was no significant difference between ueSRT and eSRT (*P* = 0.6171, Fig. 1). Furthermore, there was a stronger outcome difference when comparing ueSRT + eSRT and dSRT (*P* = 0.0108, Fig. 2), whereas no significant difference was observed when comparing ueSRT and eSRT + dSRT (*P* = 0.2991, Fig. 3).

Univariate analysis revealed that the presence of Gleason pattern 5 (including tertiary pattern 5), lymphovascular invasion, perineural invasion, timing of SRT (ueSRT vs eSRT vs dSRT; and ueSRT + eSRT vs dSRT) and PSA doubling time <6 months were associated with poorer failure-free survival (Table 2). Of the two covariates regarding the timing of SRT, we only included “ueSRT + eSRT and dSRT” (a stronger one) but not “ueSRT vs eSRT vs dSRT” (a weaker one) in the subsequent multivariate model, because these factors should be closely-correlated and would therefore cancel out each other’s statistical significance (i.e. multicollinearity). PSA doubling time was also excluded from the model, since it was only calculable in 51 (67 %) patients. Accordingly, multivariate analysis identified the presence of Gleason pattern 5, perineural invasion and dSRT (reference: ueSRT + eSRT) as independent predictors of failure of SRT (Table 2).

**Table 2:** Univariate and multivariate analyses of clinicopathological factors for failure-free survival

Parameter	Cutoff	Univariate	Multivariate	
		*P*	HR (95 % CI)	*P*
Age	≥65 years vs <65 years†	0.2151		
Initial PSA	≥20 ng/mL vs <20 ng/mL	0.9923		
Pathological T stage	≥T3 vs ≤ T2	0.5521		
Pathological GS	≥8 vs ≤7	0.0558		
Gleason pattern 5	Yes vs no	0.0004*	4.166 (1.351 to 12.76)	0.0133*
Extraprostatic extension	Yes vs no	0.4370		
Lymphovascular invasion	Yes vs no	0.0101*	1.005 (0.323 to 3.130)	0.9935
Positive surgical margin	Yes vs no	0.2967		
Seminal vesicle invasion	Yes vs no	0.1506		
Perineural invasion	Yes vs no	0.0157*	5.876 (1.139 to 107.5)	0.0316*
Timing of SRT	ueSRT vs eSRT vs dSRT	0.0364*	–	–‡
	ueSRT + eSRT vs dSRT	0.0108*	3.117 (1.141 to 7.880)	0.0280*
	ueSRT vs eSRT + dSRT	0.2991		
SRT technique	3DCRT vs IMRT	0.4964		
Total dose	≥66 Gy vs <66 Gy	0.6278		
Concomitant ADT	Yes vs no	0.3118		
PSA doubling time	≥6 months vs <6 months	0.0071*	–	–‡

*HR* hazard ratio, *CI* confidence interval, *PSA* prostate-specific antigen, *GS* Gleason score, *SRT* salvage radiotherapy, *ueSRT* ultra-early salvage radiotherapy, *eSRT* early salvage radiotherapy, *dSRT* delayed salvage radiotherapy, *3DCRT* 3-dimensional conformal radiation therapy, *IMRT* intensity-modulated radiotherapy, *ADT* androgen deprivation therapy

†median; *statistically significant; ‡these covariates were excluded from the multivariate analysis (see text)

Concerning the treatment modality, although univariate analysis did not demonstrate a significant difference between 3DCRT vs IMRT, IMRT exhibited a non-significant trend for better failure-free survival than 3DCRT: 2 of 15 (13 %) patients who underwent IMRT failed biochemically, whereas 18 of 61 (30 %) who received 3DCRT did.

With regard to the secondary endpoints, two patients (3 %) in the dSRT group developed clinical metastases: One developed a solitary metastasis to a right iliac lymph node but has achieved undetectable PSA with subsequent salvage ADT; and another developed multiple metastases to bone and para-aortic lymph nodes and finally died of PC. No other patient than the latter one died from any cause during the follow-up period.

## Discussion

The present study compared outcomes of different timings of SRT for post-RP BCR, especially focusing on the prognostic significance of ueSRT. During the follow-up period (median: 70 months), four of 20 (20 %), nine of 40 (23 %) and seven of 16 (44 %) patients failed biochemically in the ueSRT, eSRT and dSRT groups. No survival benefit of ueSRT compared to eSRT was demonstrated in the study, whereas dSRT was associated with worse prognosis as previously reported. These results thus support the current recommendations that SRT should be performed after two consecutive PSA values ≥0.2 ng/mL and before reaching 0.5 ng/mL [9–13].

To the best of our knowledge, no previous study has investigated the effects of SRT given before patients meet the current definition of BCR. Although we previously demonstrated the benefit of ultra-early salvage ADT for post-RP BCR in pT2-4 N0 patients [8], this result was not reproducible for ueSRT. This difference may be attributable to the difference between systemic ADT and local radiation therapies, though the reason currently remains unclear.

Several studies have associated SRT given at a pre-radiation PSA ≤0.5 ng/mL with better outcomes [9–13], although current guidelines only recommend a pre-radiation PSA <1.0 ng/mL [3, 4]. For example, Briganti et al. demonstrated that eSRT given before PSA reached 0.5 ng/mL achieved an equivalent oncological outcome to adjuvant radiotherapy in patients with pT3N0 PC, as stated above [10]. Several clinical trials comparing the outcomes of adjuvant radiotherapy and eSRT are now underway to clarify this issue (RAVES; EORTC 22043-30041; GETUG-17) [11, 15].

Pathological Gleason pattern 5, including tertiary pattern 5, was an independent predictor of failure-free survival in this study. Some previous studies also reported that Gleason pattern 5 was a strong prognostic factor in patients undergoing salvage treatments (SRT and/or ADT) for post-RP BCR [16, 17]. Gleason pattern 5 may thus be a more critical marker than conventional Gleason grading system in this setting.

Perineural invasion was also identified as an independent predictor of failure-free survival. Several studies have reported perineural invasion as a significant prognostic factor in the setting of SRT for post-RP BCR [18–21]. Our results are in complete accord with these previous studies.

With regard to the treatment modality, although univariate analysis did not demonstrate a significant difference between 3DCRT vs IMRT, those who underwent IMRT (*n* = 15) had a relatively favorable outcome: Only 2 of 15 (13 %) patients failed biochemically. As new technologies such as IMRT and/or image-guided radiotherapy have improved outcomes of external beam radiotherapy [22], its evaluations should be updated often.

Our study had several limitations. The ueSRT group may have included patients without evidence of BCR, which may have resulted in overestimation of the outcomes of ueSRT. Other limitations were its retrospective design, small sample size, selection bias and lead-time bias. Randomized prospective studies with longer follow-up periods are thus needed to confirm these preliminary results.

## Conclusions

No survival benefit of ueSRT was shown in patients with post-RP BCR, whereas dSRT was associated with poorer outcome as reported in previous studies. These results may validate the current recommendations that SRT should be performed after two consecutive PSA values ≥0.2 ng/mL and before reaching 0.5 ng/mL.

## Abbreviations

3DCRT, 3-dimensional conformal radiation therapy; ADT, androgen deprivation therapy; BCR, biochemical recurrence; dSRT, delayed salvage radiotherapy; eSRT, early salvage radiotherapyvGS, Gleason score; IMRT, intensity-modulated radiotherapy; IQR, interquartile range; PC, prostate cancer; PSA, prostate-specific antigen; RP, radical prostatectomy; SRT, salvage radiotherapy; ueSRT, ultra-early salvage radiotherapy

## References

[1] Hull GW, Rabbani F, Abbas F, Wheeler TM, Kattan MW, Scardino PT (2002). Cancer control with radical prostatectomy alone in 1,000 consecutive patients. J Urol.

[2] Roehl KA, Han M, Ramos CG, Antenor JA, Catalona WJ (2004). Cancer progression and survival rates following anatomical radical retro-pubic prostatectomy in 3,478 consecutive patients: long-term results. J Urol.

[3] Thompson IM, Valicenti RK, Albertsen P, Davis BJ, Goldenberg SL, Hahn C, Klein E, Michalski J, Roach M, Sartor O, Wolf JS, Faraday MM (2013). Adjuvant and salvage radiotherapy after prostatectomy: AUA/ASTRO Guideline. J Urol.

[4] Freedland SJ, Rumble RB, Finelli A, Chen RC, Slovin S, Stein MN, Mendelson DS, Wackett C, Sandler HM, American Society of Clinical Oncology (2014). Adjuvant and salvage radiotherapy after prostatectomy: American Society of Clinical Oncology clinical practice guideline endorsement. J Clin Oncol.

[5] Mohler JL, Kantoff PW, Armstrong AJ, Bahnson RR, Cohen M, D’Amico AV, Eastham JA, Enke CA, Farrington TA, Higano CS, Horwitz EM, Kane CJ, Kawachi MH, Kuettel M, Kuzel TM, Lee RJ, Malcolm AW, Miller D, Plimack ER, Pow-Sang JM, Raben D, Richey S, Roach M 3rd, Rohren E, Rosenfeld S, Schaeffer E, Small EJ, Sonpavde G, Srinivas S, Stein C, Strope SA, Tward J, Shead DA, Ho M; National Comprehensive Cancer Network. NCCN Clinical Practice Guidelines in Oncology (NCCN Guidelines®) Prostate Cancer Version 2.2014. Fort Washington: National Comprehensive Cancer Network, Inc.; 2014.

[6] Mir MC, Li J, Klink JC, Kattan MW, Klein EA, Stephenson AJ (2014). Optimal definition of biochemical recurrence after radical prostatectomy depends on pathologic risk factors: identifying candidates for early salvage therapy. Eur Urol.

[7] Hirao Y (2012). Clinical Practice Guidelines for Prostate Cancer: The Japanese Urological Association 2012 update.

[8] Taguchi S, Fukuhara H, Azuma T, Suzuki M, Fujimura T, Nakagawa T, Ishikawa A, Kume H, Igawa Y, Homma Y (2014). Ultra-early versus early salvage androgen deprivation therapy for post-prostatectomy biochemical recurrence in pT2-4N0M0 prostate cancer. BMC Urol.

[9] Stephenson AJ, Scardino PT, Kattan MW, Pisansky TM, Slawin KM, Klein EA, Anscher MS, Michalski JM, Sandler HM, Lin DW, Forman JD, Zelefsky MJ, Kestin LL, Roehrborn CG, Catton CN, DeWeese TL, Liauw SL, Valicenti RK, Kuban DA, Pollack A (2007). Predicting the outcome of salvage radiation therapy for recurrent prostate cancer after radical prostatectomy. J Clin Oncol.

[10] Briganti A, Wiegel T, Joniau S, Cozzarini C, Bianchi M, Sun M, Tombal B, Haustermans K, Budiharto T, Hinkelbein W, Di Muzio N, Karakiewicz PI, Montorsi F, Van Poppel H (2012). Early salvage radiation therapy does not compromise cancer control in patients with pT3N0 prostate cancer after radical prostatectomy: results of a match-controlled multi-institutional analysis. Eur Urol.

[11] Pfister D, Bolla M, Briganti A, Carroll P, Cozzarini C, Joniau S, van Poppel H, Roach M, Stephenson A, Wiegel T, Zelefsky MJ (2014). Early salvage radiotherapy following radical prostatectomy. Eur Urol.

[12] Ploussard G, Staerman F, Pierrevelcin J, Larue S, Villers A, Ouzzane A, Bastide C, Gaschignard N, Buge F, Pfister C, Bonniol R, Rebillard X, Fadli S, Mottet N, Saint F, Saad R, Beauval JB, Roupret M, Audenet F, Peyromaure M, Delongchamps NB, Vincendeau S, Fardoun T, Rigaud J, Soulie M, Salomon L, Committee of Cancerology (CCAFU) of the Association of French Urology (AFU) (2014). Clinical outcomes after salvage radiotherapy without androgen deprivation therapy in patients with persistently detectable PSA after radical prostatectomy: results from a national multicentre study. World J Urol.

[13] Kwon O, Kim KB, Lee YI, Byun SS, Kim JS, Lee SE, Hong SK (2014). Salvage radiotherapy after radical prostatectomy: prediction of biochemical outcomes. PLoS One.

[14] Roberts SG, Blute ML, Bergstralh EJ, Slezak JM, Zincke H (2001). PSA doubling time as a predictor of clinical progression after biochemical failure following radical prostatectomy for prostate cancer. Mayo Clinic Proc.

[15] Pearse M, Fraser-Browne C, Davis ID, Duchesne GM, Fisher R, Frydenberg M, Haworth A, Jose C, Joseph DJ, Lim TS, Matthews J, Millar J, Sidhom M, Spry NA, Tang CI, Turner S, Williams SG, Wiltshire K, Woo HH, Kneebone A (2014). A Phase III trial to investigate the timing of radiotherapy for prostate cancer with high-risk features: background and rationale of the Radiotherapy -- Adjuvant Versus Early Salvage (RAVES) trial. BJU Int.

[16] Song C, Kim YS, Hong JH, Kim CS, Ahn H (2010). Treatment failure and clinical progression after salvage therapy in men with biochemical recurrence after radical prostatectomy: radiotherapy vs androgen deprivation. BJU Int.

[17] Jackson W, Hamstra DA, Johnson S, Zhou J, Foster B, Foster C, Li D, Song Y, Palapattu GS, Kunju LP, Mehra R, Feng FY (2013). Gleason pattern 5 is the strongest pathologic predictor of recurrence, metastasis, and prostate cancer-specific death in patients receiving salvage radiation therapy following radical prostatectomy. Cancer.

[18] Do T, Parker RG, Do C, Tran L, Do L, Dolkar D (1998). Salvage radiotherapy for biochemical and clinical failures following radical prostatectomy. Cancer J Sci Am.

[19] De Meerleer G, Fonteyne V, Meersschout S, Van den Broecke C, Villeirs G, Lumen N, Ost P, Vandecasteele K, De Neve W (2008). Salvage intensity-modulated radiotherapy for rising PSA after radical prostatectomy. Radiother Oncol.

[20] Ost P, De Troyer B, Fonteyne V, Oosterlinck W, De Meerleer G (2011). A matched control analysis of adjuvant and salvage high-dose postoperative intensity-modulated radiotherapy for prostate cancer. Int J Radiat Oncol, Biol, Phys.

[21] Ost P, Lumen N, Goessaert AS, Fonteyne V, De Troyer B, Jacobs F, De Meerleer G (2011). High-dose salvage intensity-modulated radiotherapy with or without androgen deprivation after radical prostatectomy for rising or persisting prostate-specific antigen: 5-year results. Eur Urol.

[22] Dolezel M, Odrazka K, Zouhar M, Vaculikova M, Sefrova J, Jansa J, Paluska P, Kohlova T, Vanasek J, Kovarik J (2015). Comparing morbidity and cancer control after 3D-conformal (70/74 Gy) and intensity modulated radiotherapy (78/82 Gy) for prostate cancer. Strahlenther Onkol.

